# Postoperative Atrial Fibrillation in Adults with Obstructive Sleep Apnea Undergoing Coronary Artery Bypass Grafting in the RICCADSA Cohort

**DOI:** 10.3390/jcm11092459

**Published:** 2022-04-27

**Authors:** Yüksel Peker, Henrik Holtstrand-Hjälm, Yeliz Celik, Helena Glantz, Erik Thunström

**Affiliations:** 1 Department of Pulmonary Medicine, Koc University Research Center for Translational Medicine [KUTTAM], Istanbul 34450, Turkey; yecelik@ku.edu.tr; 2 Division of Sleep and Circadian Disorders, Brigham and Women’s Hospital and Harvard Medical School, Boston, MA 02115, USA; 3 Department of Molecular and Clinical Medicine, Institute of Medicine, Sahlgrenska Academy, University of Gothenburg, 40530 Gothenburg, Sweden; henrik.holtstrand.hjalm@vgregion.se (H.H.-H.); erik.thunstrom@vgregion.se (E.T.); 4 Department of Clinical Sciences, Respiratory Medicine and Allergology, School of Medicine, Lund University, 22185 Lund, Sweden; 5 Division of Pulmonary, Allergy, and Critical Care Medicine, University of Pittsburgh School of Medicine, Pittsburgh, PA 15213, USA; 6 Department of Internal Medicine, Skaraborg Hospital, 53151 Lidköping, Sweden; helena.glantz@vgregion.se

**Keywords:** coronary artery disease, coronary artery bypass grafting, atrial fibrillation, obstructive sleep apnea

## Abstract

Postoperative atrial fibrillation (POAF) occurs in 20–50% of patients with coronary artery disease (CAD) after coronary artery bypass grafting (CABG). Obstructive sleep apnea (OSA) is also common in adults with CAD, and may contribute to POAF as well to the reoccurrence of AF in patients at long-term. In the current secondary analysis of the Randomized Intervention with Continuous Positive Airway Pressure (CPAP) in Coronary Artery Disease and Obstructive Sleep Apnea (RICCADSA) trial (Trial Registry: ClinicalTrials.gov; No: NCT 00519597), we included 147 patients with CABG, who underwent a home sleep apnea testing, in average 73 ± 30 days after the surgical intervention. POAF was defined as a new-onset AF occurring within the 30 days following the CABG. POAF was observed among 48 (32.7%) patients, occurring within the first week among 45 of those cases. The distribution of the apnea-hypopnea-index (AHI) categories < 5.0 events/h (no-OSA); 5.0–14.9 events/h (mild OSA); 15.0–29.9 events/h (moderate OSA); and ≥30 events/h (severe OSA), was 4.2%, 14.6%, 35.4%, and 45.8%, in the POAF group, and 16.2%, 17.2%, 39.4%, and 27.3%, respectively, in the no-POAF group. In a multivariate logistic regression model, there was a significant risk increase for POAF across the AHI categories, with the highest odds ratio (OR) for severe OSA (OR 6.82, 95% confidence interval 1.31–35.50; *p* = 0.023) vs. no-OSA, independent of age, sex, and body-mass-index. In the entire cohort, 90% were on β-blockers according to the clinical routines, they all had sinus rhythm on the electrocardiogram at baseline before the study start, and 28 out of 40 patients with moderate to severe OSA (70%) were allocated to CPAP. During a median follow-up period of 67 months, two patients (none with POAF) were hospitalized due to AF. To conclude, severe OSA was significantly associated with POAF in patients with CAD undergoing CABG. However, none of those individuals had an AF-reoccurrence at long term, and whether CPAP should be considered as an add-on treatment to β-blockers in secondary prevention models for OSA patients presenting POAF after CABG requires further studies in larger cohorts.

## 1. Introduction

Atrial fibrillation (AF) is the most common cardiac arrhythmia affecting up to 33% of general populations [1,2]. AF is associated with hypertension, coronary artery disease (CAD), cardiomyopathies, and increases the risk for ischemic stroke and systemic embolism [2]. Moreover, the traditionally recognized risk factors for AF are also risk factors for ischemic stroke [3,4]. Many CAD patients require coronary artery bypass grafting (CABG) surgery, and postoperative atrial fibrillation (POAF) has been reported in 20–50% of those individuals [2,5,6,7]. Though many episodes of POAF are known to be self-terminating, there have been reports suggesting that POAF may increase the risk for recurrent AF in the next five years [8]. Moreover, it has been shown that POAF may be a risk factor for stroke, myocardial infarction, and mortality compared with non-POAF patients following cardiac and non-cardiac surgery [9,10,11]. Fatal embolic events have been proposed as the main contributing factor for the increased mortality risk in patients with POAF [12]. Other complications of POAF have been referred to prolonged hospital stay and increased health-care consumption [13,14].

Obstructive sleep apnea (OSA), being characterized by intermittent partial or complete collapse of the upper airways during sleep (hypopneas/apneas), leads to intermittent episodes of hypoxemia, hypercapnia, sympathetic activity, arousals, and intrathoracic pressure swings, altogether affecting normal physiology [15]. These changes are associated with increased inflammatory activity and endothelial dysfunction as well as remodeling of the left atrium, which in turn increases the risk of AF [16]. Unrecognized severe OSA has been related with new-onset AF in non-cardiac surgery [17]. It has also been shown that patients with OSA are at an increased risk for POAF [18,19], and readmission within 30 days following the CABG surgery [20]. In a questionnaire-based study, a high probability of OSA at baseline was found to be a significant predictor of POAF [21]. Moreover, obesity, closely linked with OSA, has also been related to the POAF [22].

To date, there is a lack of research evidence regarding a possible interaction between OSA and POAF, and whether it has influence on reoccurrence of AF and long-term adverse cardiovascular outcomes in patients undergoing CABG.

The Randomized Intervention with CPAP in CAD and OSA (RICCADSA) trial primarily addressed the impact of CPAP on the composite of repeat revascularization, myocardial infarction, stroke, and cardiovascular mortality in revascularized patients with CAD and OSA [23,24]. In the current study, we analyzed the prevalence of POAF in a subgroup of patients from the RICCADSA cohort, who had undergone CABG, and addressed the association between POAF and OSA, and its possible impact on the reoccurrence of AF and long-term adverse cardiovascular outcomes.

## 2. Materials and Methods

### 2.1. Study Population

**Figure 1:**
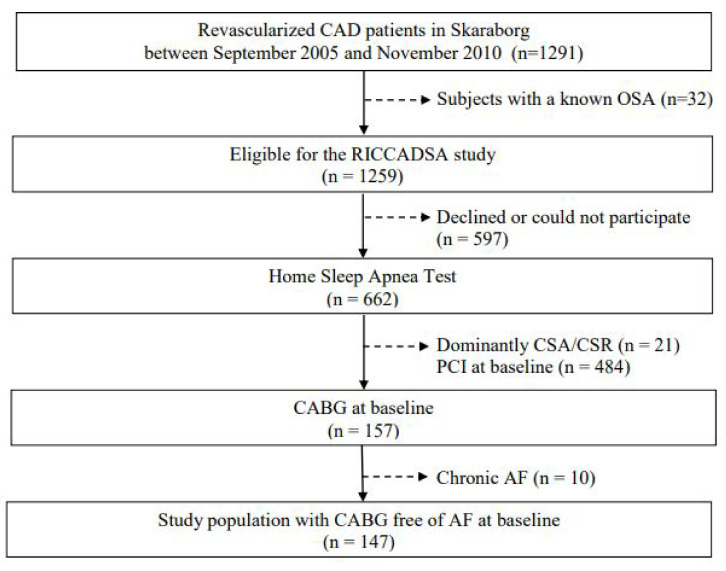
Consort flow chart of the analytic study sample. Definition of abbreviations: AF = atrial fibrillation; CAD = coronary artery disease; CABG = coronary artery bypass grafting; CSA/CSR = Central Sleep Apnea/Cheyne-Stokes Respiration; PCI = percutaneous coronary intervention; POAF = Postoperative atrial fibrillation; RICCADSA = Randomized Intervention with Continuous Positive Airway Pressure in Coronary Artery Disease and Obstructive Sleep Apnea.

The study design and methods of the RICCADSA trial have been published previously [23]. In brief, the RICCADSA cohort consisted of adults with CAD, who underwent revascularization (percutaneous coronary intervention (PCI) or CABG) in Skaraborg County, West Sweden, and investigated by a home sleep apnea test (HSAT) in a stable condition following the revascularization procedure. The patients were recruited between December 2005 and November 2010, and the final follow-up was in May 2013. In the parent trial, the CAD patients moderate to severe OSA (apnea –hypopnea-index (AHI) ≥ 15 events/h) who had no excessive daytime sleepiness (Epworth Sleepiness Scale (ESS) score < 10) were randomized to CPAP or no-CPAP, the ones with the excessive sleepiness (ESS score ≥ 10) were offered CPAP. The CAD patients without OSA (AHI < 5/h) were included in the observational arm and followed prospectively [21]. Patients with dominantly central sleep apnea/Cheyne–Stokes respiration (CSA/-CSR) were excluded. For the purpose of the current study, only patients with CABG at baseline and no history of AF before the CABG procedure (*n* = 147) were included in the cross-sectional analysis of the baseline cohort (Figure 1).

### 2.2. Definition of Comorbidities

While the timeframe of POAF is not strictly defined in literature, it has been usually considered within a week after surgery with peak incidence between postoperative day 2 and 4 [2]. However, it has been shown that patients with OSA are at increased risk for readmission within 30 days following the CABG surgery [20], and we have therefore defined POAF as a new-onset AF within 30 days after the CABG for the current study. The POAF was detected by continuous electrocardiography telemetry during the initial postoperative care and by repeated electrocardiograms at the follow up visits. Body mass index (BMI) was calculated (body weight in kilograms divided my height in meters squared). Obesity was defined as BMI ≥ 30 kg/m^2^ [25]. Current smoking was defined as current habitual smoking for at least 6 months at the time of the study start. Lung disease included chronic obstructive lung disease or asthma at baseline. Patients were labelled as hypertensive if they either had a hypertension diagnosis, and/or were receiving antihypertensive treatment. The ESS questionnaire was used to evaluate subjective excessive daytime sleepiness [26] and allocation of the patients to the randomized controlled arm or the observational arm of the main trial [23]. The ESS contains eight questions to evaluate the chance of dozing off under eight scenarios in the past month. Each item is scored from 0 to 3 (0 for would never doze, 1 for slight chance of dozing, 2 for moderate chance of dozing, and 3 for high chance of dozing). The ESS score ranges from 0 to 24. Excessive daytime sleepiness was defined as an ESS score of ≥10 as previously described [23]. Anthropometrics, smoking habits, medical history of the study population, as well as medications were obtained from the medical records.

### 2.3. Sleep Studies, Group Allocation

On average, the ambulatory HSAT was performed 73 ± 30 days after the CABG procedure. The portable, HSAT was conducted with the Embletta^®^ Portable Digital System device (Embla, Broomfield, CO, USA), and consisted of the following tools: (1) nasal pressure detector using nasal cannula/pressure transducer system; (2) thoraco-abdominal movement detection through two XactTrace™ inductive belts with respiratory inductance plethysmography technology; (3) finger pulse oximeter detecting heart rate and oxyhemoglobin saturation (SpO_2_); and (4) body position and movement detection. The sleep time was estimated on the basis of self-reporting as well as the pattern of body movement during the HSAT. Apneas were defined as an almost complete (≥90%) cessation of airflow. Hypopneas were defined as a ≥50% reduction in thoraco-abdominal movement and/or a ≥50% decrease in the nasal pressure amplitude for ≥10 s [25]. In addition, the total number of significant oxyhemoglobin desaturations (decrease of ≥4% from the immediately preceding baseline) were scored, and the oxygen desaturation index (ODI) was calculated as the number of significant desaturations per hour of estimated sleep. Additionally, time spent below 90% saturation (T90%) was recorded. Events with a ≥30% reduction in thoraco-abdominal movement and/or a ≥30% decrease in the nasal pressure amplitude for ≥10 s were also scored as hypopneas when there was a significant desaturation (≥4%) [27]. The reference group was the CAD patients with an AHI < 5.0 events/h, i.e., no-OSA. The widely used cut-offs for mild, moderate, and severe OSA are AHI 5.0 and 14.9 events/h; AHI 15.0–29.9 events/h, and AHI ≥ 30 events/h, respectively [28]. Mild OSA cases after screening with HSAT were included in the current protocol for baseline associations but not in the long-term follow-up as they were excluded from the main RICCADSA trial in order to avoid “overlapping” cases for OSA vs. no-OSA [23].

The 1:1 randomization of the participants with CAD and nonsleepy OSA in the main trial was scheduled with a block size of eight patients (four CPAP, four controls) stratified by sex and type of revascularization (PCI/CABG) [21,22]. The nonsleepy participants with OSA who were randomized to CPAP and the ones with sleepy OSA were fitted with an auto-adjusting device (S8^®^ or S9^®^; ResMed, Sydney, Australia) by trained staff. Additional details of the follow-ups, including CPAP adherence, were published previously [24,29].

### 2.4. Blood Sampling

All blood samples were collected in EDTA and serum tubes on the morning following the baseline sleep recordings in the parent RICCADSA trial. As described previously [30], plasma N-terminal-prohormone of brain natriuretic peptide (p-NT-proBNP) levels were measured using the commercially available solid-phase 2-site chemiluminescent enzyme-labeled immunometric assay on an Elecsys system (Roche Diagnostics; Mannheim, Germany) on samples obtained from 2005 to 2007, and on an Immulite 2000 XPi (Siemens Healthcare Diagnostics, Cardiff, UK) from 2008 to 2010.

### 2.5. Transthoracic Echocardiography

As previously described in detail [30,31], cardiac function was assessed on the same day of the study following the collection of the blood samples. Echocardiographic examinations were conducted by experienced echocardiography technicians according to the study hospital’s clinical practice on a commercially available cardiac ultrasound system (Vivid-7 General Electric Healthcare, Fairfield, CT, USA). Images were stored and evaluated with a commercially available software program (EchoPAC General Electric Healthcare). All examinations were evaluated by the same offline examiner (HG) who was unaware of the patients’ clinical and sleep data. Left atrial diameter was measured on parasternal M-mode images as the linear distance between the trailing edge of the posterior aortic wall and the leading edge of the posterior wall. An overall evaluation of the left ventricular ejection fraction (LVEF) was performed by visual estimation, and when appropriate, by the Simpsons biplane method [30,31].

### 2.6. Statistical Analysis

For descriptive statistics, means and standard deviations were reported for continuous variables, and counts with percentages were given for categorical variables. Shapiro–Wilk test was used to test normality assumption of the current data for all variables. The baseline differences between the patients with POAF vs. no-POAF were tested by independent-sample T-test or Mann–Whitney U when appropriate for the continuous data, and by the Chi-square test for the categorical data. A binary logistic regression analysis was performed to determine the variables associated with POAF. Age, sex, obesity, and OSA severity were entered into the multivariate model with additional adjustments for the significant variables in the univariate analyses. All statistical tests were two-sided, odds ratios (ORs) with 95% confidence interval (CI) were reported, and a *p*-value < 0.05 was considered significant. Statistical analyses were performed using IBM Corp ^®^ Released 2019. IBM SPSS Statistics for Windows, Version 26.0 (IBM Corp, Armonk, NY, USA).

### 2.7. Outcomes and Sample Size

The main outcome of the current protocol was the occurrence of POAF in patients undergoing CABG, and its association with OSA as well as reoccurrence of AF and long-term outcomes in terms of hospitalization due to AF and/or cardiac failure. The clinical follow-up data were obtained from the patients’ medical charts as well as from the Swedish Hospital Discharge Registry.

The sample size estimation for the main RICCADSA trial was based on the estimates for the primary endpoints, and no specific power estimate was established for the current post-hoc analysis.

## 3. Results

### 3.1. The Entire Study Population and Participants at Follow-Up

**Figure 2:**
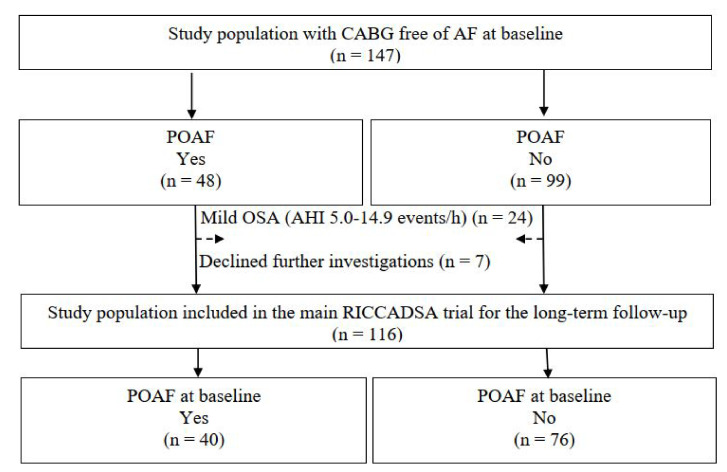
Consort flow chart for the follow-up sample. Definition of abbreviations: AF = atrial fibrillation; AHI = apnea hypopnea index; CABG = coronary artery bypass grafting; POAF = Postoperative atrial fibrillation; RICCADSA = Randomized Intervention with Continuous Positive Airway Pressure in Coronary Artery Disease and Obstructive Sleep Apnea.

Among the 147 participants of the RICCADSA cohort who underwent CABG, 48 patients (32.7%) had POAF (Figure 2). HSAT was conducted in average 73 ± 30 days after the CABG surgery.

**Figure 3:**
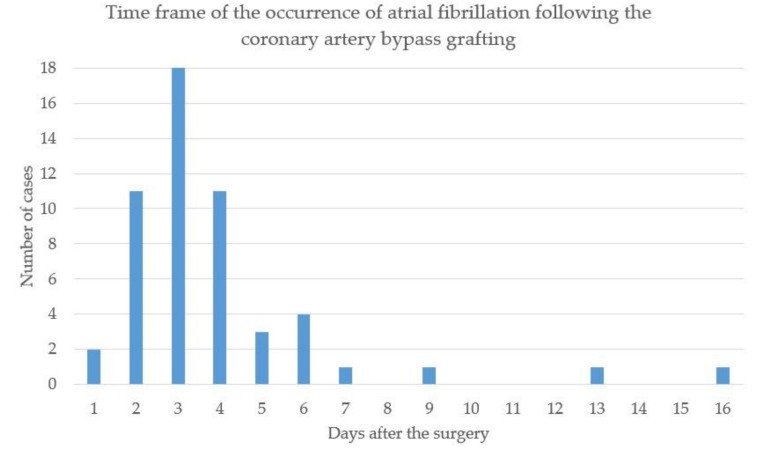
Time frame of the occurrence of POAF in 48 cases following the surgery.

As shown in Table 1, baseline demographic and clinical characteristics did not differ significantly between the patients with vs. without POAF. Almost all POAF cases were observed within seven days, except three cases occurring on days 9, 13, and 16, respectively (Figure 3). The β blocker use was similar in both groups. The proportion of patients without OSA was 4.2% in the POAF group vs. 14.1% in the no-POAF group, whereas severe OSA was observed among 45.8% of the patients with POAF compared to 27.3% in the no-POAF group (*p* = 0.025) (Table 1).

### 3.2. Occurrence of POAF and Its Association with OSA

**Figure 4:**
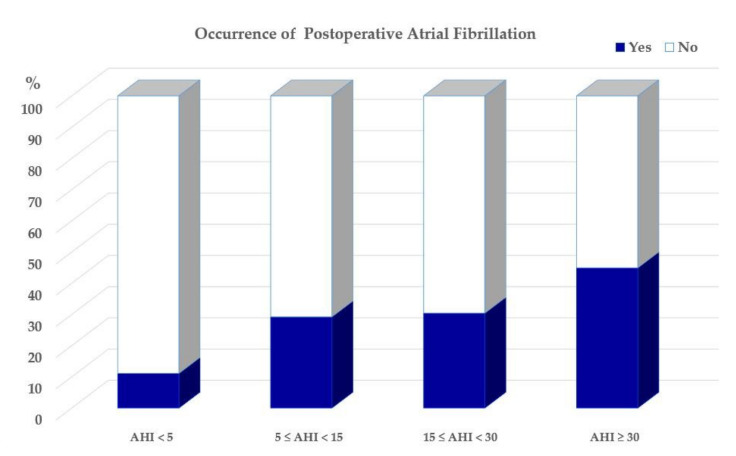
Proportion of occurrence of POAF across the AHI categories. Definition of abbreviations: AF = atrial fibrillation; AHI = apnea hypopnea index.

As illustrated in Figure 4, the distribution of the occurrence of POAF was 11.1%, 29.2%, 30.4%, and 44.9%, respectively, across the OSA severity categories.

As shown in Table 2, age, AHI, ODI, and severe OSA were significantly associated with the occurrence of POAF in the univariate analyzes. There was a trend towards statistical significance for LAD and the circulating p-NT-proBNP values, but other demographic and clinical characteristics were not associated with the occurrence of POAF. In a multivariate logistic regression model, there was a significant risk increase for POAF across the AHI categories with the highest OR for severe OSA (OR 6.82, 95% CI 1.31–35.50; *p* = 0.023) vs. no-OSA, independent of age, sex, and body-mass-index.

### 3.3. Long-Term Outcomes

Among the 116 patients included in the main RICCADSA trial, 38 out of the 40 patients with POAF (95.2%) had moderate to severe OSA, of whom 28 (70%) were allocated to CPAP at baseline. At the one-year follow-up, 12 (42.9%) were using the device at least 4 h/night corresponding all nights. During a median follow-up of 67 months, only two patients (none with POAF at baseline) were hospitalized due to AF.

## 4. Discussion

The main findings of the current study included that almost one third of the patients undergoing CABG in the RICCADSA cohort demonstrated POAF, which is in line with the existing literature. We found that severe OSA, defined as an AHI of at least 30 events/h was independently associated with POAF. Notwithstanding, none of the patients with POAF at baseline had a new onset of AF, which required a hospital admission during a median follow-up of 67 months. 

As aforementioned, AF is the most common cardiac arrhythmia affecting up to 33% of general populations [1,2]. AF is strongly related with hypertension, CAD, cardiomyopathies, ischemic stroke, and systemic embolism [3]. Moreover, POAF has been reported in 20–50% of CAD patients undergoing CABG [2,5,6]. In many cases, the episodes of POAF are self-terminating, but there have also been reports suggesting that POAF may increase the risk for recurrent AF in the next five years [8], and may be a risk factor for stroke, myocardial infarction, and mortality compared with non-POAF patients following cardiac and non-cardiac surgery [9,10,11]. Fatal embolic events [12] as well as prolonged hospital stay and increased health-care consumption [13,14] have also been reported. 

It is widely known that obstructive events during sleep lead to intermittent episodes of hypoxemia, hypercapnia, sympathetic activity, arousals, and intrathoracic pressure swings, altogether affecting normal physiology [15]. These changes are also associated with increased inflammatory activity and endothelial dysfunction as well as remodeling of the left atrium, which in turn increases the risk of AF [16]. 

OSA has previously been found to be a risk factor of readmissions to hospital postoperatively [20,32]. In line with the previous studies, our results show that severe OSA is associated with AF, when compared to non-OSA patients, and with patients with no or mild to moderate OSA. In a meta-analysis by Qaddoura et al. [19], OSA patients had a two-fold risk increase in for POAF. Similar results were reported in a later meta-analysis by Nagappa et al. [33]. 

AF has been shown to be the most common of postoperative complications with associated sequelae [34]. In a study population consisting of almost 300,000 CABG patients, Jawitz et al. showed that new-onset AF was found in 30% during follow up, almost two and half times as common compared to the second most common complication (prolonged ventilatory support), and over six times as common as the third most common complication (renal failure) [35]. Similar results for POAF were reported in an earlier and smaller study conducted by Aranki et al. [36]. OSA is considered to lead to cardiac remodeling [37], and may therefore be involved in the development of AF. Thus, identifying and treating OSA may lead to reduce the adverse cardiovascular outcomes.

Interestingly, none of the patients who had POAF at baseline demonstrated reoccurrence during the follow-up period, which might be related with the fact that 90% of the entire cohort were on treatment with β-blockers at baseline before the start of the RICCADSA trial, and 70% of the OSA patients were allocated to CPAP. 

### Limitations of the Study

The small sample size of this post-hoc analysis of the CABG subgroup is the main limitation of the study, and the results should therefore be interpreted cautiously. We should also acknowledge that the patients were not screened for AF after discharge from the hospital. AF is often asymptomatic [1,38], and the reoccurrence of AF could therefore be missed during the follow-up period. Another limitation refers to the generalizability of the findings since the RICCADSA trial was a single-center, two-site study, and the results may not be valid for other geographic regions and races and other types of cardiac surgery. 

## 5. Conclusions

Our results showed that severe OSA was significantly associated with POAF in patients with CAD undergoing CABG, of whom 90% were on β-blockers and 70% were allocated to CPAP treatment at the initiation of the study. None of the patients with the POAF history at baseline had reoccurrence of AF that required long-term hospitalization. Whether or not CPAP should be considered as an add-on treatment to β-blockers in secondary prevention models for OSA patients presenting POAF after CABG requires further studies in larger cohorts.

## Figures and Tables

**Table 1:** Baseline characteristics of the entire study population (*n* = 147).

	POAF *n* = 48	No POAF *n* = 99
Age *, yrs	66.5 ± 7.5	63.1 ± 8.7
Male sex, %	89.6	84.8
BMI, kg/m^2^	28.0 ± 4.5	27.7 ± 4.1
Obesity %	22.9	24.2
AHI categories *, %		
<5.0 events/h (no OSA)	4.2	16.2
5.0–14.9 events/h (mild)	14.6	17.2
15.0–29.9 events/h (moderate)	35.4	39.4
≥30.0 events/h (severe)	45.8	27.3
ESS ≥ 10, %	37.5	32.3
Current smoking, %	4.2	14.1
Hypertension, %	64.6	61.2
Diabetes, %	33.3	21.2
Stroke, %	4.2	11.2
Lung disease, %	8.3	8.1
Diuretic use, %	34.3	30.9
β blocker use, %	89.2	89.7
Aspirin use, %	80.0	95.8
Clopidogrel use, %	4.6	1.5
Warfarin use, %	13.7	1.5
CCB use, %	18.2	17.0
ACE inhibitor use, %	34.3	37.2
ARB use, %	11.4	7.8
Lipid-lowering agent use, %	93.7	97.5
**Echocardiography** ^†^	***n* = 39**	***n* = 75**
LAD *, mm	45.6 ± 5.9	43.4 ± 5.7
LVEF %	54.8 ± 8.4	56.9 ± 5.0
p-NT-proBNP, ng/mL	705.2 ± 1164.5	419.3 ± 416.5

Continuous variables are expressed as median and boundaries of interquartile ranges. Definition of abbreviations: ACE = angiotensin-converting enzyme; AHI = apnea–hypopnea index, ARB = angiotensin II receptor blocker; BMI = body mass index; CABG = Coronary artery bypass grafting; CCB = calcium channel blocker; ESS = Epworth Sleepiness Scale; LAD = left atrium diameter; LVEF = left ventricular ejection fraction; p-NT-proBNP = plasma N-terminal-prohormone of brain natriuretic peptide; POAF = Postoperative atrial fibrillation; RICCADSA = Randomized Intervention with Continuous Positive Airway Pressure in Coronary Artery Disease and Obstructive Sleep Apnea. ^†^ No data from the mild OSA group. * *p* < 0.05.

**Table 2:** Unadjusted ORs (95% CIs) for variables associated with POAF.

	OR	Lower	Upper	*p* Value
Age, years	1.05	1.01	1.01	0.024
Male sex	1.54	0.52	4.51	0.435
BMI, kg/m^2^	1.02	0.94	1.10	0.690
Obesity	0.93	0.41	2.10	0.860
Current smoking	0.26	0.06	1.21	0.087
Hypertension	1.16	0.56	2.37	0.694
Diabetes	1.86	0.86	4.01	0.115
Lung disease	1.03	0.30	3.62	0.958
AHI, events/h	1.03	1.01	1.05	0.003
ODI, events/h	1.04	1.01	1.06	0.007
T90%, %	1.01	0.99	1.02	0.545
AHI categories				
<5.0 events/h	1			
5.0–14.9 events/h	3.29	0.59	18.27	0.173
15.0–29.9 events/h	3.49	0.72	16.87	0.105
≥ 30 events/h	6.52	1.35	31.46	0.020
LAD, mm	1.07	0.99	1.14	0.068
LVEF %	1.05	0.98	1.11	0.157
p-NT-proBNP, pg/mL	1.00	1.00	1.00	0.090

Definition of abbreviations: AHI = apnea–hypopnea index, CI = confidence inetrval; LAD = left atrium diameter; LVEF = left ventricular ejection fraction; ODI = oxygen desaturation index; OR = odds ratio; p-NT-proBNP = plasma N-terminal-prohormone of brain natriuretic peptide; POAF = Postoperative atrial fibrillation; T90% = Time spent below 90% oxygen saturation.

## Data Availability

Individual participant data that underlie the results reported in this article can be obtained by contacting the principal investigator of the RICCADSA trial; yuksel.peker@lungall.gu.se.
